# An Investigation into the Immunomodulatory Activities of *Sutherlandia frutescens* in Healthy Mice

**DOI:** 10.1371/journal.pone.0160994

**Published:** 2016-08-30

**Authors:** Wei Lei, Jimmy D. Browning, Peggy A. Eichen, William R. Folk, Grace Y. Sun, Dennis B. Lubahn, Kevin L. Fritsche

**Affiliations:** 1 Division of Animal Sciences, University of Missouri, Columbia, Missouri, United States of America; 2 Department of Biochemistry, University of Missouri, Columbia, Missouri, United States of America; 3 Department of Nutrition and Exercise Physiology, University of Missouri, Columbia, Missouri, United States of America; Katholieke Universiteit Leuven Rega Institute for Medical Research, BELGIUM

## Abstract

*Sutherlandia frutescens* is a medicinal plant that has been traditionally used in southern Africa for cancers, infections, and inflammatory conditions. We recently published experiments demonstrating that an aqueous extract of *S*. *frutescens* possessed potent immune-stimulatory activity. This work was carried out with murine macrophages, an immune cell type that plays a pivotal role in host defense from infection and in shaping host inflammatory and immune responses. Here, we conducted a series of follow-up experiments to explore the impact of consuming *S*. *frutescens* on host response to bacterial challenge using healthy mice. We found that feeding mice a diet containing *S*. *frutescens* failed to significantly alter host response to systemic infection by either a gram-positive or gram-negative bacterium (i.e., *L*. *monocytogenes* and *E*. *coli*, respectively). In contrast to the *in vitro* observations, we found no evidence that *S*. *frutescens* consumption stimulated *in vivo* inflammatory responses; instead, consumption of *S*. *frutescens* tended to diminish *in vivo* inflammatory responses. Several possible reasons for this are discussed.

## Introduction

Recently we used a murine macrophage cell line and primary murine macrophages to demonstrate that extracts of *S*. *frutescens* have potent immune-stimulatory activities.[1] Specifically, short-term treatment with a crude aqueous extract of *S*. *frutescens* or with a polysaccharide-enriched fraction of *S*. *frutescens* increased macrophage production of reactive oxygen species (ROS) and nitric oxide (NO). ROS are a group of highly reactive free radical and non-radical molecules, including superoxide (O_2_•_-_), hydrogen peroxide (H_2_O_2_), hydroxyl radicals (OH•), hypochloric acid (HOCl), and peroxynitrite (ONOO-).[2] NO has been recognized as a versatile mediator of immune responses.[3] Both ROS and NO play critical roles in host defense to infection.[4–6] Additionally, the aqueous extract and polysaccharides-enriched fractions of *S*. *frutescens* activated NF-κB signaling in murine macrophages resulting in an increased production of TNFα.[1] TNF-α is a pro-inflammatory cytokine that plays an important role in host defense.[7, 8] Our findings as well as the work of others are consistent with a large body of research indicating that numerous botanical polysaccharides possess immune-stimulatory activities, most often via actions on innate immune cells, such as macrophages. [9]

Importantly, for many botanicals the evidence of their immune-modifying activity is limited to actions on isolated immune cells (i.e., *in vitro*). While such data may illuminate possible molecular and cellular mechanisms through which extracts might enhance the functioning of a host’s immune system, evidence of *in vivo* immune-modifying activity is lacking.

Traditional uses of *S*. *frutescens* are generally by oral consumption of the vegetative parts in the form of teas or decoctions, or as a dietary supplement in the form of capsules or tablets. Accordingly, we sought to investigate the potential immune-modulating activity of this medicinal plant following oral consumption. Based on our previous *in vitro* observations with extracts of *S*. *frutescens* and the research conducted by others, we hypothesized that oral consumption of this botanical would enhance host defense against infection from *Listeria monocytogenes* (EGD strain). *L*. *monocytogenes* is a gram-positive bacterium that causes sepsis and meningitis in immuno-compromised patients and a serious maternal/fetal infection in pregnant women.[10, 11] In light of the key role that macrophages play in controlling the early host response to this pathogen [12] and our prior observation that extracts of *S*. *frutescens* enhanced NO and ROS generation in vitro, we expected that mice fed *S*. *frutescens* would have significantly fewer *L*. *monocytogenes* in their phagocyte-rich organs (i.e., spleen and liver) compared to control mice.

Therefore, we carried out a series of feeding trials with healthy mice that were provided experimental diets, identical except for the presence of 0, 0.25 and 1% (wt/wt) of *S*. *frutescens*. After several weeks of the dietary intervention mice were challenged with live *L*. *monocytogenes* or *E*. *coli* and host clearance of these bacteria as well as sickness behavior were assessed. Additionally, we measured the impact of consuming *S*. *frutescens* on in vivo and ex vivo production of pro-inflammatory mediators (e.g., cytokines and chemokines) in an effort to better understand how this botanical might influence in vivo host innate immune and inflammatory responses.

## Materials and Methods

### Reagents

Lipopolysaccharide (from *E*. *coli* 026:B6 or *E*. *coli* 0111:B4) was purchased from Sigma-Aldrich (St. Louis, MO, USA). Pam2CSF (tlrl-pm2s-1) was purchased from InvivoGen (San Diego, CA, USA). *E*. *coli* (K12) and *L*. *monocytogenes* (EGD strain, BAA-679) were obtained from American Type Culture Collection (ATCC). The bead-based cytokines and chemokines Multi-Plex kit (Cat. MCYTOMAG-70K-PMX) was obtained from EMD Millipore (Billerica, MA, USA). The diet ingredients were purchased from Dyets, Inc (Bethlehem, PA, USA).

### Animal care

All animal-based elements described in this research were approved by the Animal Care and Use Committee of the University of Missouri-Columbia. C57BL/6 or BALB/c female and male mice (~4-week old) were purchased from Jackson Laboratory (Bar Harbor, Maine, USA). Mice were housed in pairs of the same sex in polycarbonate cages (W-L-D: 11.5 × 7.5 × 5″). Mice were kept in an animal facility that maintained an environmental temperature of 23 ± 2°C with a 12:12 h light:dark cycle. At all times, mice had free access to food and distilled water. The health and general wellbeing of the mice were assessed daily, and body weights were measured weekly (daily during bacteria challenge portions of these studies). After a 7-day acclimation period, mice were randomly assigned into three dietary treatment groups: 0, 0.25 or 1.0% (wt/wt) ground *S*. *frutescens* leaf powder in AIN-93G basal rodent diet. [13]

### Experimental diets

The botanical-containing diets were made using the ground powder of the vegetative parts of *Sutherlandia frutescens* purchased from Big Tree Nutraceutical (Fish Hoek, South Africa) and authenticated as described previously.[1] According to previous reports, the most commonly used dose of *S*. *frutescens* in humans is 2.5 g dry materials (leaf) per day.[14] For a 70 kg adult human, this dose of *S*. *frutescens* intake would be equivalent to 35.7 mg/kg per day. Therefore, we used 36 mg/kg per day to calculate the human equivalent dose for experimental diets in our mouse feeding studies. The human equivalent dose was calculated using a formula that adjusts for differences in metabolic body size and is described in detail elsewhere.[15, 16] For mice this dose was estimated to be 411 mg/kg body weight per day. The average body weight of our healthy adult mice was ~25 g, therefore we estimated that each mouse would need to consume 11 mg *S*. *frutescens* to achieve the dosage of 411 mg/kg BW per day. Food consumption for these mice was expected to be 4–5 g/d based on our previous experience and reports in the literature, thus to deliver ~11 mg of *S*. *frutescens* per day, the diets should contain approximately 0.25% (g/100 g) of the ground *S*. *frutescens* material. We also prepared a diet containing four times this dosage (i.e., 1.0%) of *S*. *frutescens* to account for unexpected and unknown differences between mice and humans relative to the uptake and metabolism of putative bioactive compounds in *S*. *frutescens*. All diets were nutritionally complete[13] (i.e., AIN-93G diets) and identical, except for the presence of *S*. *frutescens* and the small amount of cornstarch that was removed to account for the addition of this botanical (**Table 1**).

**Table 1:** Composition of Experimental Diets Containing 0, 0.25 or 1.0% *S*. *frutescens* (SF) for Mouse Feeding Experiments (g/kg).

Ingredients^a^	0% SF (control)	0.25% SF	1.0% SF
Cornstarch	397.0	395.0	387.0
Casein	200.0	200.0	200.0
Dextrose	132.0	132.0	132.0
Sucrose	100.0	100.0	100.0
Fiber (cellulose)	50.0	50.0	50.0
Mineral mix (AIN-93)	35.0	35.0	35.0
Vitamin mix (AIN-93G)	10.0	10.0	10.0
L-Cystine	3.0	3.0	3.0
Choline bitartrate	2.5	2.5	2.5
Soybean oil	70.0	70.0	70.0
Food dye (color varies) ^b^	0.0	0.2	0.2
***S*. *frutescens* (SF)** ^c^	**0.0**	**2.5**	**10.0**

a Ingredients for AIN-93G based rodent diets were purchased from Dyets, Inc., Bethlehem, Pennsylvania, United States of America, unless otherwise noted below.

b Sensient Technologies, St. Louis, Missouri, United States of America.

c Ground powder of vegetative parts of *S*. *frutescens* (L.) R. Br. (Big Tree Nutraceutical, Fish Hoek, South Africa)

### *In vivo* bacterial challenges

To assess the impact of dietary *S*. *frutescens* on host-resistance from infection by *L*. *monocytogenes* we used healthy male and female BALB/c. This strain of mice is known to be susceptible to infection from *L*. *monocytogenes*.[17] After 4 weeks of consuming the experimental diets, all mice received an intravenous injection with ~10^4^ cfu of *L*. *monocytogenes* (EGD strain) in 0.2 mL of sterile, endotoxin-free phosphate-buffered saline.

To investigate the impact of dietary *S*. *frutescens* on host response to an *E*. *coli* infection, male and female C57BL/6 mice were used. After consuming one of the experimental diets for 4 weeks, mice were injected intraperitoneally with ~10^8^ colony-forming units (*cfu*) of *E*. *coli* (K-12 strain) in 1 mL of sterile PBS.

Following bacterial challenge, mice were monitored twice daily for clinical symptoms of illness using a standard scoring sheet designed for monitoring and recording pain/discomfort in rodents and approved by the Animal Care and Use Committee of the University of Missouri. Any mice showing signs of pain or distress (e.g., ruffled fur, shallow breathing, hunched back) were immediately and humanely euthanized. As expected, no mice in our experiments experienced clinical symptoms requiring premature termination, however, we did record three premature deaths during the *E*. *coli* challenge experiments (i.e., female mice in CONTROL and 0.25% SF groups and a male mouse in the 1.0% SF group) and one unexpected death during the *L*. *monocytogenes* experiments (i.e., a female mouse in the 0.25% SF group).

### Sickness behavior

The impact of *S*. *frutescens* on spontaneous locomotor activity before and during an infection was measured by mini-emitters as described by Spiers et al.[18] Representative mice (n = 4 of each sex) within each diet treatment group were lightly anesthetized and mini-emitters were surgical implanted subcutaneously along the spine just below the scapulas. Following a 4-day post-surgery recovery period, activity data were recorded every 5 seconds with accumulated events for every 20 min period for a 3-day period prior to and 3 days or 36 hours following the challenge with *L*. *monocytogenes* or *E*. *coli*, respectively, following the bacterial challenge.

### Measurement of host clearance of the bacterial challenge

We determined the number of *L*. *monocytogenes* present in the spleen and liver of mice 3 days following the challenge. This timeframe best reflects the capacity of the innate immune system to control the cell-to-cell spread and proliferation of this intracellular pathogen.[19, 20] Therefore, 3 days following the challenge with *L*. *monocytogenes* mice were humanely killed and the liver and spleen from each mouse was aseptically collected and weighed. One hundred milligram portions of each tissue were transferred to 1.5 mL sterile centrifuge tubes containing 0.2 mL of sterile PBS and 100 mg of 0.5mm zirconium oxide beads. Tubes were kept on ice until processed for bacterial load determination as described below.

The bacterial load in the liver and spleen was determined by a fluorescence-based microplate assay.[21] The assay is based on the reduction of the non-fluorescent dye resazurin to a fluorescent product, resorufin, by bacterial enzyme activity. Liver and spleen samples were collected and then homogenized using a Bullet Blender^TM^ (Next Advance, Inc., New York, United States of America) according to the manufacturer’s instructions and described elsewhere.[22] One hundred microlitersof the homogenates were diluted to 1.0 mL (10-fold) with PBS containing 0.2% saponin, then 7 μL of these diluted homogenates were added to wells of a black 384 well plate in triplicate. Each well contained 50 μL of 0.01% resazurin in LB medium. Resorufin fluorescence was measured every 20 minutes for 10 hours. The time required to reach 8000 RFU (T_8K_) was determined for each sample and cfu/mg tissue calculated using the standard curve. A suspension containing 1x10^9^ cfu/mL of *L*. *monocytogenes* or *E*. *coli* was prepared, and serial 10-fold dilutions of this suspension were done in ice-cold LB medium containing 0.01% resazurin to create standards in the range 1x10^2^ to 1x10^8^ *cfu*/mL. Fifty microliters of these standards were added in triplicate to a pre-chilled black 384 well plate and fluorescence (excitation, 530 nm; emission, 590 nm) was read every 20 min for 10 h using a fluorescence plate reader (BioTek Synergy HT). A standard curve was then constructed that relates the log of the *cfu*/well to the time required to reach a standard fluorescence of 8000 relative fluorescence units (RFU). The limit of detection for this assay was approximately 4x10^3^ *cfu*/g tissue. For liver samples that were below the limit of detection in the fluorescence assay, cfu/mg tissue was calculated using a traditional microbiological plate count approach. For these liver samples only, 100 μL of the diluted homogenate was plated on TSAY (tryptic soy agar + yeast extract) plates. Plates were incubated overnight at 37°C and colonies counted manually the next day.

### *In vivo* host inflammatory response

In order to assess the impact of *S*. *frutescens* consumption on host inflammatory response, BALB/c and C57BL/6 mice were fed with experimental diets as described previously, followed by an intraperitoneal injection with *L*. *monocytogenes* (EGD strain, 5 x 10^6^ *cfu*/mouse) and LPS (from *E*. *coli* 026:B6, 20 μg/mouse), respectively. Mice were anesthetized 2 hr post-LPS injection or 24 hrs. after *L*. *monocytogenes* challenge and blood was drawn by cardiac puncture. After allowing the blood to clot on ice for 30 minutes, serum was separated by centrifugation (1000× g, 20 min.) and stored at -80°C for later analyses of inflammatory cytokines/chemokines as described below.

### Isolation and stimulation of peritoneal macrophages

Thioglycolate-elicited peritoneal macrophages were isolated from healthy mice as described previously.[23] Crude (i.e., unfractionated) peritoneal cells were re-suspended in Dulbecco's Modified Eagle's Medium (DMEM) with 5% FBS, and seeded into a 96-well plate at a density of 1×10^5^ cells/well. Cells were cultured in the incubator at 37°C with 5% CO_2_ for 3 h. The non-adherent cells were removed by washing with PBS, leaving behind a monolayer of macrophages (typically > 95% purity).

We used a commercial synthetic diacylated lipoprotein (i.e., Pam2CSK, Invivogen, San Diego, CA, USA) to mimic *L*. *monocytogenes* infection for these ex vivo experiments, while LPS served to model *E*. *coli* infection. The production of inflammatory cytokines/chemokines by elicited cells was also evaluated. We conducted dose-response experiments to determine the optimal concentration for each of these inflammatory stimuli in our assay. The concentrations used (i.e., 5 ng of Pam2CSK or 100 ng of LPS per mL of culture medium) provided a robust, but sub-maximal stimulation. Primary macrophages were co-cultured with LPS or Pam2CSK for 24 h, and the cell culture medium was harvested, stored at -80°C for later cytokines and chemokines quantitation.

### Analysis of the inflammatory cytokines and chemokines

The concentrations of inflammatory cytokines and chemokines in cell culture medium or in mouse serum were determined using bead-based Multi-Plex kits (Cat. MCYTOMAG-70K-PMX, EMD Millipore, Billerica, MA, USA). The standard kit allowed for the quantitation of 25 analytes: granulocyte-colony stimulating factor (G-CSF), granulocyte/monocyte-CSF (GM-CSF), interferon (IFN)-γ, interleukin-1(IL-1)α, IL-1β, IL-2, IL-3, IL-4, IL-5, IL-6, IL-7, IL-9, IL-10, IL-12p40, IL-12p70, IL-13, IL-15, IL-17A, interferon-induced protein (IP)-10 (CXCL10), keratinocyte chemoattractant (KC, CXCL3), monocyte chemoattractant protein (MCP)-1 (CCL2), macrophage inflammatory protein (MIP)-1α (CCL3), MIP-1β (CCL4), MIP-2α (CXCL2), regulated on activation, normal T cell expressed and secreted (RANTES, CCL5) and tumor necrosis factor (TNF)-α. Depending on the specific experiment, however, kits with a more limited analyte profile were purchased to save money and to focus on those analytes of greatest interest. Briefly, samples were diluted as recommended or as needed by 2-, 10-, or 100-fold using DMEM medium or a serum-matrix diluent (provided by the kit manufacturer). Twenty-five microliters of diluted samples were transferred into a black 96-well plate, and incubated with antibody beads overnight at 4°C with shaking. Beads were immobilized using a magnet and the liquid was removed, followed by two washes. Then the plate with beads was incubated with the detection antibody for 1 h, followed by streptavidin-phycoerythrin for 30 min. After the supernatant was removed, 150 μL of drive fluid was added to each well, and the plate was read using the MAGPIX^®^ plate reader with xPONENT software (4.2 Luminex, Austin, Texas, United States of America). Data were analyzed using the MILLIPLEX™ analyst software.

### Statistical analyses

All data were analyzed by one-way analysis of variance (ANOVA) using SAS 9.3 software (SAS Institute Inc., Cary, NC, USA). The post-comparisons were performed using Tukey’s multiple comparison tests. A *p < 0*.*05* was considered to indicate statistical significance. Data are presented as means ± SEM, except on those occasions when unequal variances existed between independent trials or assays, then LSmeans ± pooled SEM are reported.

## Results

### Host resistance to bacterial infection

After consuming diets with 0.25% or 1.0% *S*. *frutescens* for ~4 weeks, the average body weight of female and male mice (both BALB/c and C57BL/6 strains) were not significantly different within sexes from that of control mice (see S1 and S2 Tables, respectively). Challenging mice with *L*. *monocytogenes* resulted in a loss of 5 to 10% of their body weight during the next few days, regardless of which experimental diet the mice were consuming. Similarly, consumption of *S*. *frutescens* had no discernable impact on the loss of body weight in mice following an *E*. *coli* challenge. Additionally, there were no significant differences across diet treatment groups in liver or spleen weight following either bacterial challenge.

**Fig 1:**
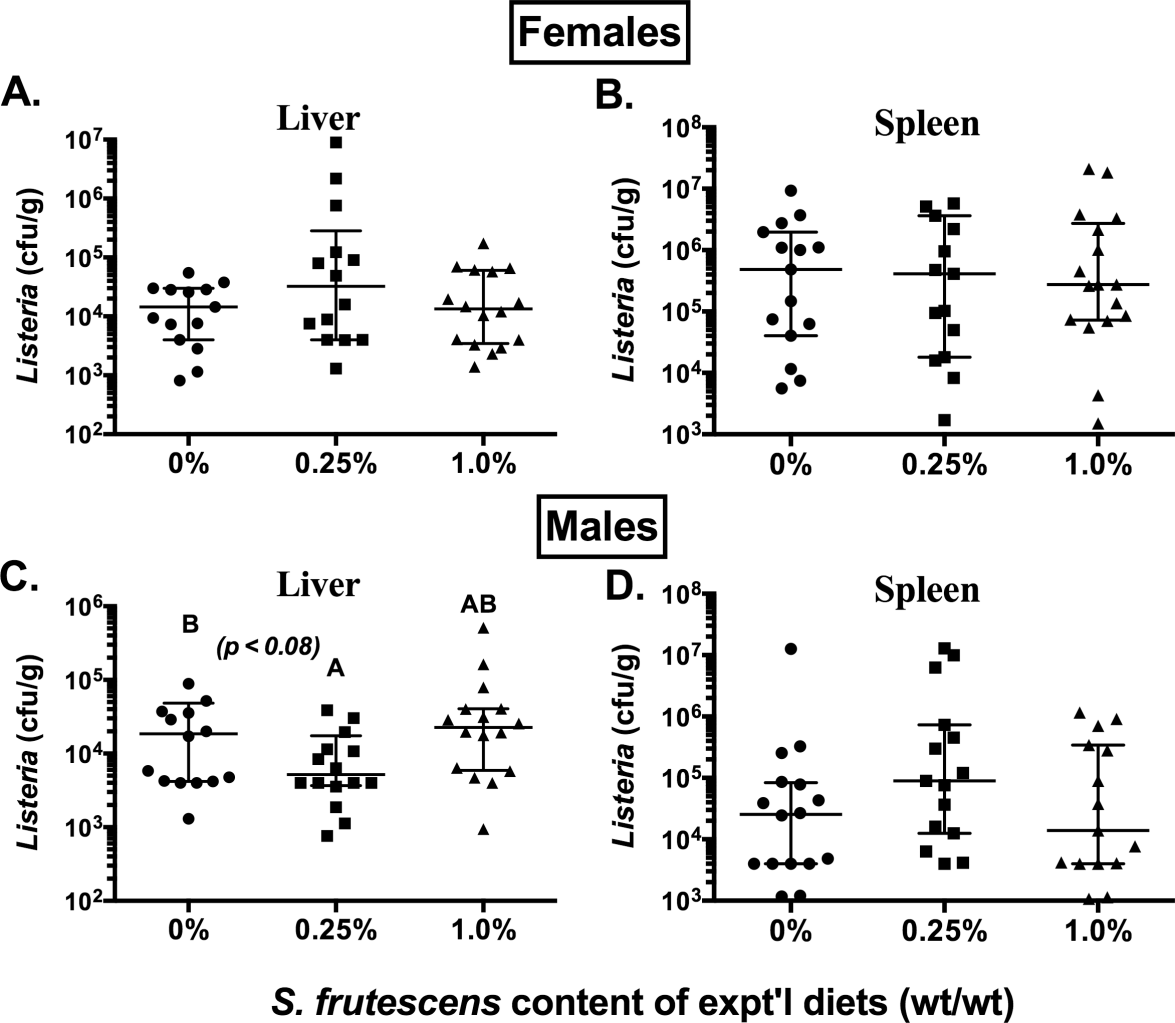
Impact of *S*. *frutescens* Consumption on Host Clearance of an Experimental Infection with *L*. *monocytogenes*. Female and male BALB/c (*L*. *monocytogenes*) mice were randomly assigned to one of three treatment groups: 0, 0.25, or 1.0% (wt/wt) *S*. *frutescens* in an AIN93G-type diet. After consuming diets for ~4 wks, all mice received an intravenous injection with ~10^4^ cfu of *L*. *monocytogenes* (EGD strain) in 0.2 mL of sterile, endotoxin-free phosphate-buffered saline. Liver and spleen samples were harvested three days after challenge and colony-forming units (*cfu*) were determined by a fluorescence-based microplate assay of tissue homogenates. Each symbol represents the data from a single mouse with the median and intra-quartile range shown.

**Fig 2:**
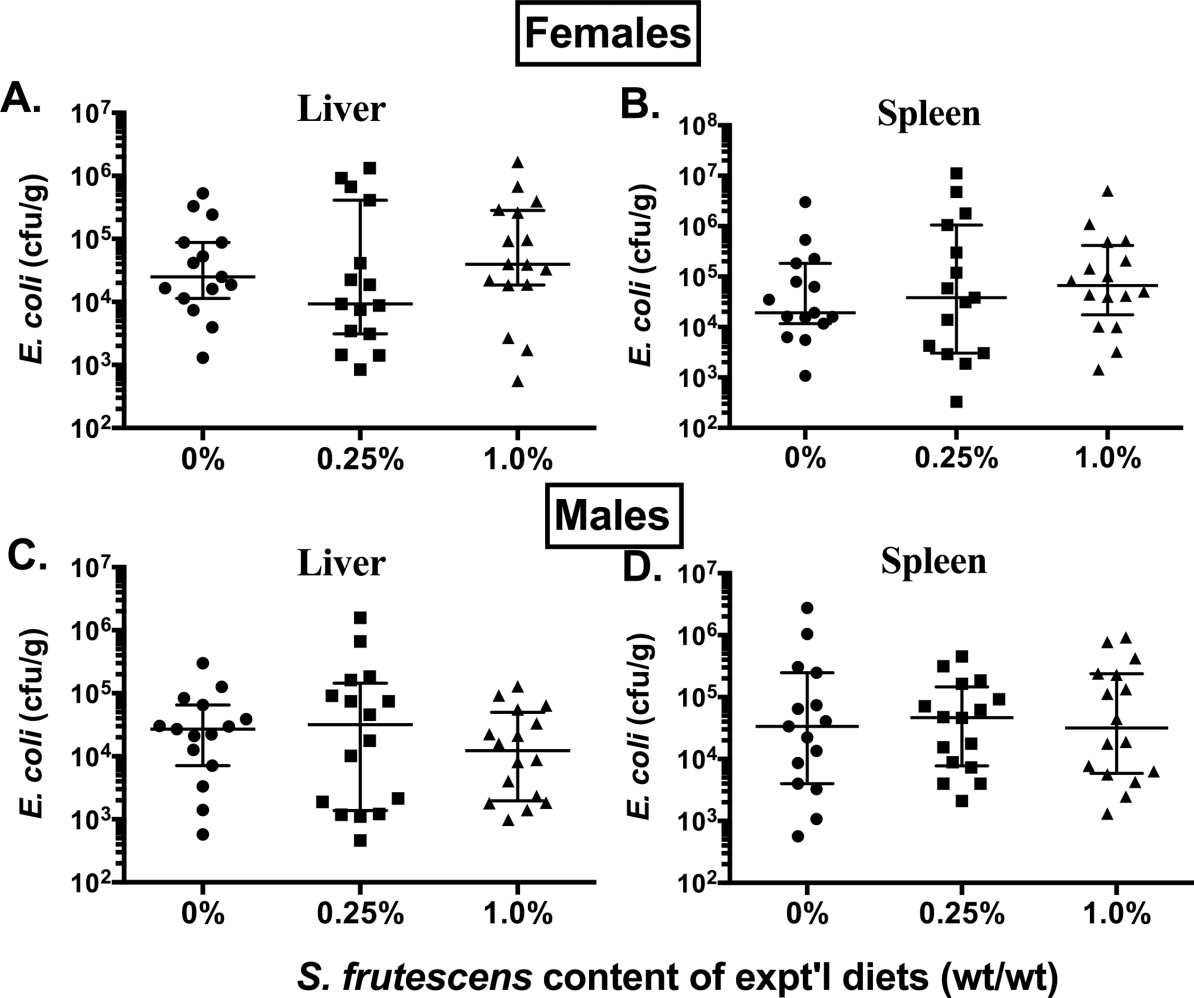
Impact of *S*. *frutescens* Consumption on Host Clearance of an Experimental Infection with *E*. *coli*. Weanling female and male C57Bl/6 mice were randomly assigned to one of three treatment groups: 0, 0.25, or 1.0% (wt/wt) *S*. *frutescens* in an AIN93G-type diet. After consuming diets for ~4 wks, all mice were injected intraperitoneally with ~10^8^ colony-forming units (*cfu*) of *E*. *coli* (K-12 strain) in 1 mL of sterile PBS. Liver and spleen samples were harvested three days after challenge and colony-forming units (*cfu*) were determined by a fluorescence-based microplate assay of tissue homogenates. Each symbol represents the data from a single mouse with the median and intra-quartile range shown.

The impact of consuming *S*. *frutescens* containing diets on host defense against infection from two commonly studied bacterial pathogens is shown in Figs 1 and 2. In contrast to our expectations, there was little indication that consumption of *S*. *frutescens* enhanced host clearance of either of these bacteria during the early stages post-challenge. We did however, note a trend (*p* < 0.08) for a modestly lower burden (~0.5 log_10_) of *L*. *monocytogenes* in the livers of male BALB/c mice fed the 0.25% *S*. *frutescens* diets compared with the mice fed the control (0% SF) or 1% *S*. *frutescens* diets (Fig 1C). Consumption of *S*. *frutescens* appeared to have no noticeable impact on the burden of *L*. *monocytogenes* in the spleens of male mice, nor on bacterial burden in the livers and spleens of the female BALB/c mice (Fig 1A, 1B and 1D). Additionally, there were no statistically significant differences in *E*. *coli* burden in livers or spleens of female or male C57BL/6 mice fed either *S*. *frutescens*-containing diets compared to the control diet (Fig 2A–2D).

### Impact of infection on spontaneous activity (i.e., sickness behavior)

**Fig 3:**
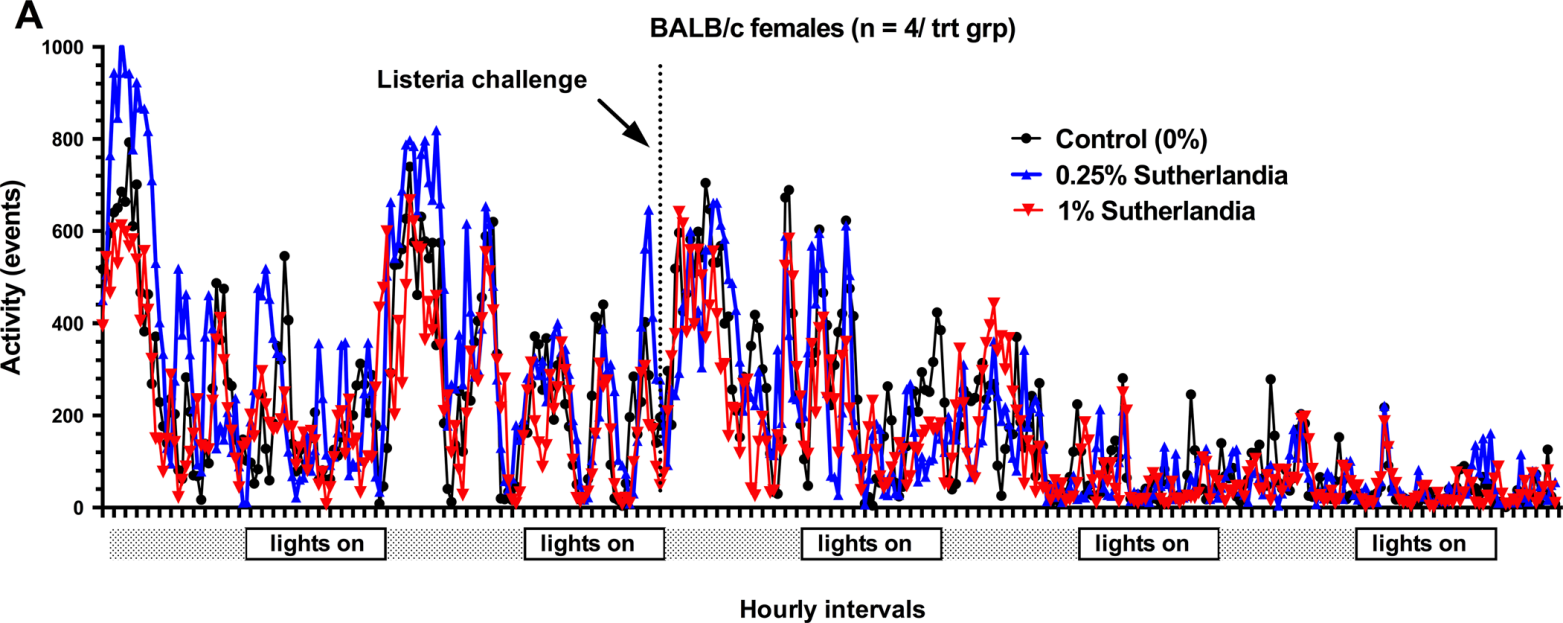
Dietary *S*. *frutescens* Has No Impact on Spontaneous Activity (i.e., sickness behavior) of Mice After an Experimental Infection with *L*. *monocytogenes*. Healthy female BALB/c weanling mice were fed experimental diets containing one of three doses of *S*. *frutescens* (i.e., 0, 0.25 or 1% by wt) for 3–4 wks. Mice were housed in pairs in a vivarium with a 12 hr light:dark cycle at a room temperature between 22–25°C and a relative humidity of 50–60% for the entirety of the study. One of each pair of mice had a mini-emitter surgically implanted under the skin along their spine between their shoulders. At ~1 week post-surgery mice received an intravenous injection of 10^4^ *cfu* of *L*. *monocytogenes*, EGD strain. Each mouse’s movement/activity was recorded every 5 seconds and reported as accumulated events for every 20 minute period. Each tick mark on the X-axis represents an hour. Data shown represent the mean activity (#events) of four mice in each diet treatment group (n = 4/diet treatment group).

**Fig 4:**
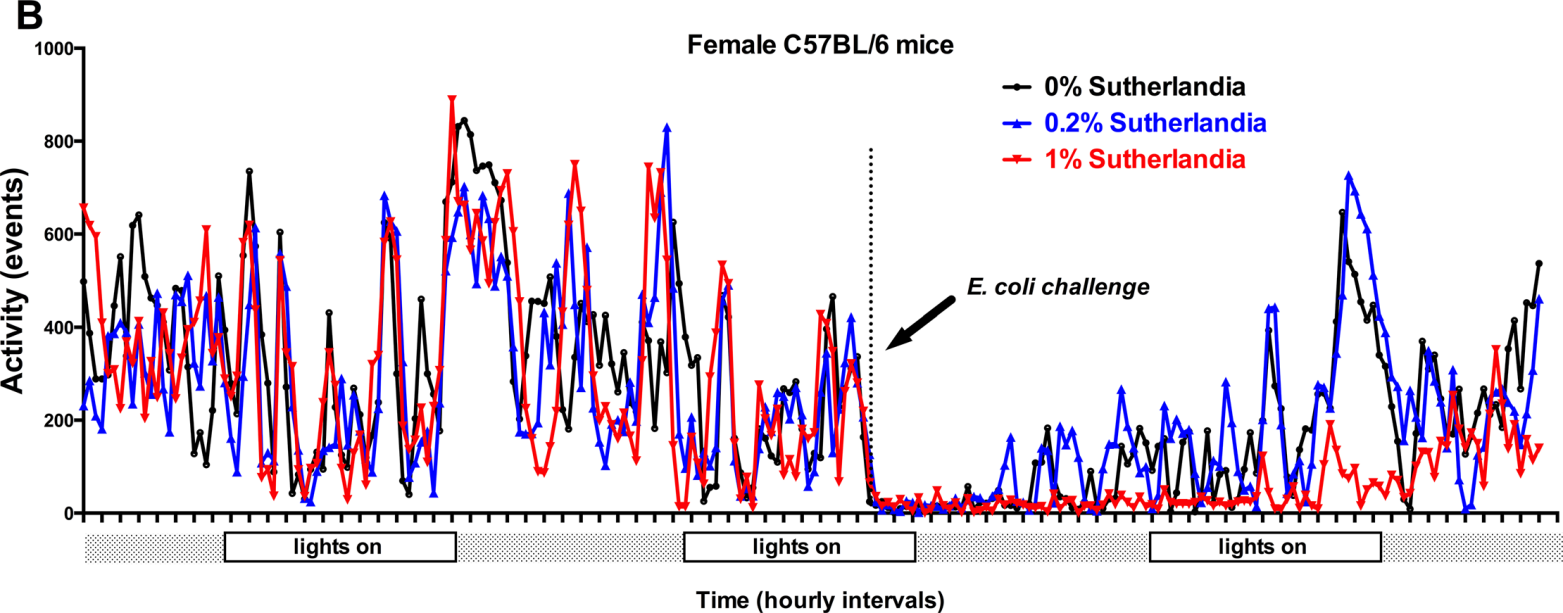
Dietary *S*. *frutescens* Has No Impact on Spontaneous Activity (i.e., sickness behavior) of Mice After an Experimental Infection with *E*. *coli*. Conditions were similar to those described in Fig 3, except in this experiment the mice were C57Bl/6 mice and the challenge consisted of an intraperitoneal injection with ~10^8^ cfu of *E*. *coli* (K-12 strain).

We implanted mini-emitters in a separate group of mice to monitor the impact of *S*. *frutescens* consumption on overall wellbeing of the mice immediately prior to and during bacterial challenges. The spontaneous activity (i.e., random locomotion within the cage) of four individual female mice in each dietary treatment group before and after infection with either *L*. *monocytogenes or E*. *coli* is shown in Fig 3 and Fig 4, respectively). Not surprisingly, spontaneous locomotor activity dramatically declined following the infectious challenge with either bacterial pathogen. Dietary *S*. *frutescens* consumption appeared to have no discernable impact on this activity either before or during the infection. While data from female mice are shown, similar results were observed for male mice similarly challenged with *L*. *monocytogenes* or *E*. *coli* (see S1 and S2 Figs).

### *In vivo* production of cytokines and chemokines

Systemic infection with *L*. *monocytogenes* in previously healthy BALB/c mice induces the production of numerous cytokines and chemokines quantified from blood samples collected 24 h post-challenge (Table 2). Monocyte chemotactic protein-1 (MCP-1, CCL2), interferon(IFN)-γ, and interleukin-6 (IL-6) were the dominant factors detected in the blood of *L*. *monocytogenes*-infected mice. We observed no significant differences in the measured cytokines and chemokines based on the experimental diet consumed prior to and 24 hours following the infectious challenge.

**Table 2:** *S*. *frutescens* (SF) Consumption Failed to Impact Circulating Cytokines and Chemokines in BALB/c Mice 24 hr Following an *in vivo* Challenge with *L*. *monocytogenes*.^*a*^

	Experimental Diet Treatments			
Analyte^b^	Control	0.25% SF	1% SF	*p*-value^c^
TNF-α	70.4 ± 4.8	73.6 ± 6.8	73.5 ± 5.4	0.82
IL-1α	978 ± 32	1006 ± 44	1026 ± 40	0.48
IL-1β	60.2 ± 4.6	66.8 ± 4.6	59.0 ± 3.8	0.39
IL-6	3134 ± 494	3494 ± 794	3490 ± 312	0.80
IL-10	34.8 ± 3.0	35.6 ± 3.6	35.4 ± 1.8	0.92
IL-12p40	32.4 ± 6.8	25.2 ± 3.4	21.6 ± 4.0	0.17
IL-12p70	246 ± 76	186 ± 22	192 ± 16	0.35
IFN-γ	4282 ± 946	3230 ± 740	3410 ± 572	0.72
GM-CSF	146 ± 6	150 ± 6	143 ± 5	0.51
MCP-1 (CCL2)	5134 ±1524	5036 ±1178	5976 ±1770	0.64
MIP-1α (CCL3)	216 ± 18	212 ± 8	210 ± 16	0.88
MIP-1β (CCL4)	424 ± 54	480 ± 56	414 ± 32	0.52
RANTES (CCL5)	228 ± 26	240 ± 24	216 ± 22	0.54
KC (CXCL1)	1140 ± 326	922 ± 198	934 ± 228	0.51
MIP-2α (CXCL2)	250 ± 6	266 ± 8	254 ± 6	0.44
IP-10 (CXCL10)	2648 ± 234	2700 ± 338	2582 ± 184	0.78

^*a*^ For this study, male BALB/c mice were fed experimental diets containing one of three doses of *S*. *frutescens* (0, 0.25 and 1% by wt) for 4 wks. After which, all mice were challenged via intraperitoneal injection with ~5 x10^6^ colony-forming units (cfu) of *L*. *monocytogenes* (EGD strain) in 1 mL of sterile PBS. Blood samples were collected 24 h following challenge, then following sera isolation specific cytokines and chemokines concentrations were determined using a commercial multiplex beads-based assay system. Data shown are from six mice from each diet treatment group (n = 6/trt); all values are means ± SEM, expressed in pg/mL. Values for G-CSF were beyond the detection limit of the kit, while IL-4 values were consistently below the MDC, thus neither were reported here. Values for T-cell derived cytokines (i.e., IL-2, IL-5, IL-7, IL-9, IL-13, IL-15, and IL-17) tended to be very low in concentration; were not significantly impacted by the diet treatment, thus were not reported.

^*b*^ Abbreviations: GM-CSF = granulocyte/monocyte-colony stimulating factor; IFN = interferon; IL = interleukin; IP = interferon-induced protein; KC = keratinocyte chemoattractant; MCP = monocyte chemoattractant protein; MIP = macrophage inflammatory protein; RANTES = regulated on activation, normal T cell expressed and secreted; TNF = tumor necrosis factor.

^***c***^ The impact of the diet intervention/treatment was tested by ANOVA, with the main effect *p*-value shown in the 4^th^ column.

To evaluate the impact of *S*. *frutescens* intake on the inflammatory response in healthy mice we challenged them with lipopolysaccharide (LPS) from *E*. *coli*. Mice received an intraperitoneal injection with 20 μg of LPS followed by blood collection 2 hours later. We observed that consumption of *S*. *frutescens* by healthy C57BL/6 mice tended (*p* < 0.09) to reduce by nearly 50% the production of tumor necrosis factor-alpha (TNF-α) in response to a LPS challenge (Table 3). As can be seen in Table 3, however, we failed to detect any other diet-induced differences in the circulating cytokines, chemokines, or growth factors quantified. We also did not observe any significant impact of *S*. *frutescens* consumption on basal or background cytokine/chemokine levels in mice injected with vehicle (i.e., PBS) alone (see S1 Table).

**Table 3:** *S*. *frutescens* Consumption Failed to Impact Circulating Cytokines and Chemokines in C57Bl/6 Mice 2 hr Following an Injection with Lipopolysaccharide from *E*. *coli*.^*a*^

	Experimental Diet Treatments			
Analyte^b^	Control	0.25% SF	1% SF	*p*-value^c^
TNF-α	626 ± 206	347 ± 14	365 ± 47	0.09
IL-1α	1424 ± 109	1508 ± 17	1534 ± 63	0.28
IL-1β	195 ± 10	186 ± 5	177 ± 11	0.24
IL-6 ^*d*^	168 ± 18	130 ± 10	153 ± 18	0.19
IL-10	1522 ± 169	1478 ± 171	1592 ± 461	0.97
IL-12p40	185 ± 22	182 ± 13	229 ± 47	0.59
IL-12p70	154 ± 18	147 ± 11	171 ± 18	0.82
IFN-γ	33 ± 3	33 ± 2	32 ± 2	0.79
G-CSF ^*d*^	313 ± 23	267 ± 14	300 ± 19	0.37
GM-CSF	257 ± 9	260 ± 4	260 ± 11	0.22
MCP-1 (CCL2) ^*d*^	44 ± 3	41 ± 2	44 ± 3	0.59
MIP-1α (CCL3) ^*d*^	14.4 ± 0.9	12.6 ± 0.7	14.2 ± 0.7	0.29
MIP-1β (CCL4) ^*d*^	28.8 ± 1.6	25.7 ± 1.2	28.2 ± 1.0	0.26
RANTES (CCL5)	1641 ± 146	1530 ± 143	1486 ± 135	0.44
KC (CXCL1) ^*d*^	254 ± 15	260 ± 16	267 ± 17	0.66
MIP-2α (CXCL2)^*d*^	56 ± 6	49 ± 3	53 ± 3	0.36
IP-10 (CXCL10) ^*d*^	3.3 ± 0.4	3.7 ± 0.3	3.7 ± 0.4	0.35

^*a*^ For this study, male C57Bl/6 mice were fed experimental diets containing one of three doses of *S*. *frutescens* (0, 0.25 and 1% by wt) for 3–4 wks. After which, all mice were challenged via intraperitoneal injection with 20 μg of LPS from *E*. *coli* 026:B6 in 1 mL of PBS. Blood samples were collected 2 h following challenge, following sera isolation, specific cytokines and chemokines concentrations were determined using a commercial multiplex beads-based assay system. Data shown are from 12 mice from each diet group (i.e., 6 mice per diet treatment group in each of two independent trials), with values representing the means ± SEM. Values for T-cell derived cytokines (i.e., IL-2, IL-4, IL-5, IL-7, IL-9, IL-13, IL-15, and IL-17) tended to be very low in concentration; were not significantly impacted by the diet treatment, thus were not reported.

^*b*^ The impact of the diet intervention/treatment was tested in SAS by ANOVA, using contrast with interaction between the two independent trials/experiments with the main effect *p*-value shown in the 4th column.

c Abbreviations (refer to Table 2): G-CSF = granulocyte-colony stimulating factor.

^*d*^ Means reported in ng/mL.

### *Ex vivo* cytokines and chemokines production

Primary peritoneal macrophages isolated from mice fed *S*. *frutescens* produced significantly less IL-1α following stimulation with LPS compared to macrophages isolated from mice fed the control diet (S4 Table). In contrast, ex vivo production of other cytokines/chemokines by LPS- or Pam2CSK-stimulated macrophages were not significantly impacted by dietary *S*. *frutescens* (S4 and S5 Tables).

## Discussion

With most botanicals for which immune-modulating activity is claimed the evidence of their activity is limited to actions on isolated immune cells (i.e., *in vitro*). Previously, we reported that a hot-water extract made from the medicinal plant *S*. *frutescens* potently activated murine macrophages, resulting in increased production of ROS and NO.[1] Production of ROS and NO by innate immune cells are early and critical components of host defense against infection from bacterial pathogens.[4, 5] We found that this immune-stimulating activity was associated with a polysaccharide-enriched fraction of this aqueous extract. Zhang et al. [24] found that pectin-type polysaccharides isolated from *Lessertia frutescens* (an alternative name for *Sutherlandia frutescens)* stimulated innate immune cells via complement fixation. The complement cascade is a part of the host’s immune response that promotes host clearance of microbes and damaged cells. There exists a large body of research showing that numerous botanical polysaccharides possess *in vitro* immune-stimulatory activity.[9] In the present study, however, we investigated the potential benefits of unfractionated vegetative parts of this botanical in response to bacterial infection. To our knowledge this is the first investigation to examine *in vivo* and *ex vivo* host anti-bacterial activity associated with the consumption of *S*. *frutescens*.

In the present study, we found that feeding healthy mice diets containing *S*. *frutescens* had little impact on the host’s immune response to either of the bacterial pathogens we tested. In light of the results from our prior *in vitro* experiments, we designed the present experiments to focus on the early or innate elements of the host’s immune response to infection. Following the intravenous administration of *L*. *monocytogenes*, these gram-positive bacterium are rapidly taken up by phagocytes, primarily in the spleen and liver.[17] Intracellular pathogens, such as *L*. *monocytogenes*, successfully replicate within these phagocytes unless the generation of ROS and NOS are stimulated.[12] Whether and to what extent the host is able to prevent replication and spread of this intracellular pathogen during the early stages of infection (e.g., 3 days post-challenge) is highly dependent upon the production of a variety of host factors, including: TNF-α,[25] IFN-γ,[26] IL-6[27] and MCP-1[28]. Therefore, we expected to find evidence that this botanical modified the early production of these host factors and that mice fed diets containing *S*. *frutescens* would contain fewer of these bacteria. However, when we sampled the blood just 24 hours post-challenge with *L*. *monocytogenes* we found no indication that consumption of *S*. *frutescens* had altered the production of these key cytokines and chemokines. Consistent with this result was our failure to observe a significant difference in the number of *L*. *monocytogenes* bacterium in either the spleen or liver of mice fed diets containing *S*. *frutescens*.

Similar to our results examining *in vivo* host response to a *L*. *monocytogenes* infection, we found that dietary *S*. *frutescens* failed to modulate the host response to a challenge with a common gram-negative organism, *E*. *coli*. Yet, we did find some indication that consumption of *S*. *frutescens* might be having a discernable impact on *in vivo* production of a key pro-inflammatory mediator, tumor necrosis factor-α (TNF-α). When otherwise healthy mice were fed a diet containing *S*. *frutescens* they showed a trend (i.e., *p* < 0.09) to produce nearly 50% less TNF-α following an injection of *E*. *coli* endotoxin (i.e., LPS). One might expect that any treatment that reduces in vivo production of this inflammatory mediator would be associated with notable changes in sickness behavior.[29] To examine this possibility we used voluntary locomotion as a surrogate for sickness behavior as described elsewhere.[29] Yet, we did not observed any indication that *S*. *frutescens* feeding affected voluntary locomotion prior to and during an active infection from either *E*. *coli* or during an infection from *L*. *monocytogenes*. We measured many cytokines and chemokines that play important roles in shaping the early immune response to infection, yet ex vivo production of IL-1α was the only one that was significantly affected by dietary *S*. *frutescens* consumption. Recently, Africa and Smith [30] reported that treatment of co-cultures of human monocytes and endothelial cells with an aqueous extract of *S*. *frutescens* greatly diminished the production of IL-1 in response to stimulation with HIV-1 antigens. That a recent human clinical trial found that consumption of milled vegetative parts of *S*. *frutescens* in capsules might promote tuberculosis, [31] is consistent with what would be expected if host production of TNF-α and IL-1β was diminished.[32, 33]

The present study was limited in a number of ways. First, the amount of *S*. *frutescens* included in the experimental diets may have been too low to elicit an observable effect. While we sought to use human equivalent dosing calculations derived from the research literature we may have been too conservative in setting our diet inclusion levels at 0.25 and 1.0%, by weight. It is possible that inclusion of more *S*. *frutescens* may have had a greater impact on in vivo host immune/inflammatory responses. Excessive production of inflammatory mediators such as ROS, NOS, and cytokines can result in tissue damage and even death.[34, 35] Consistent with this was our preliminary findings that mice fed a diet containing 2% (wt/wt) of ground leaves from *S*. *frutescens* experienced lower survival rates (i.e., 17%, 1 of 6 mice survived) than mice fed a diet devoid of this botanical following an LD_50_ *E*. *coli* challenge.

A second limitation emerged based on the contradictory findings generated *in vitro* with this botanical. We have reported that ethanolic extracts of *S*. *frutescens* possess potent anti-inflammatory activities[36], while aqueous extracts of *S*. *frutescens* stimulate macrophage inflammatory responses.[1] Therefore, inclusion of unfractionated botanical in the diet may have failed to noticeably modify host response to bacterial challenge due to a cancelling effect of two or more components with opposite or contradictory activities. In an attempt to address this possibility, we conducted a small follow-up experiment where we administered by oral gavage either an aqueous or ethanolic extract of *S*. *frutescens* to a limited number of mice. After 7 days, half of the mice in each group were challenged with LPS or Pam2CSK and blood was collected 2 hours later. However, we found no discernible effect of either extract of *S*. *frutescens* on *in vivo* production of inflammatory cytokines and chemokines.

Third, our experimental design was limited in that we only included single time points for the assessment of bacterial clearance and circulating inflammatory cytokines and chemokines. It is possible that *S*. *frutescens* may have had a significant impact on host responses at different times after the immune challenges. Another obvious limitation of our study was that we only examined host response to a single gram-negative bacterium and single gram-positive bacterium (i.e., *E*. *coli* and *L*. *monocytogenes*, respectively). Host response to other pathogens, bacterial as well as viral, can vary tremendously. In short, our failure to observe significant modulation of host response to these two pathogens cannot exclude the possibility that treatment with *S*. *frutescens* might not provide benefits for host immunity against other infectious agents.

## Conclusions

In conclusion, this study is the first to examine whether oral consumption of unfractionated vegetative parts of *S*. *frutescens* can modify the host immune response *in vivo*. We found that host clearance of a gram-positive and a gram-negative bacterium from mice was not significantly altered by *S*. *frutescens* intake. *S*. *frutescens* exhibited only a modest impact on the production of a couple of select inflammatory cytokines. Whether such changes occur in humans, and if they do, whether they would provide clinically relevant benefits to individuals using this medicinal plant remains uncertain.

## Supporting Information

S1 Fig Impact of Dietary *S*. *frutescens* on Spontaneous Activity (i.e., sickness behavior) of Male Mice After an Experimental Infection with either *L*. *monocytogenes*. Healthy male BALB/c weanling mice were fed experimental diets containing one of three doses of *S*. *frutescens* (i.e., 0, 0.25 or 1% by wt) for 3–4 wks. Mice were housed in pairs in a vivarium with a 12 hr light:dark cycle at a room temperature between 22-25°C and a relative humidity of 50–60% for the entirety of the study. One of each pair of mice had a mini-emitter surgically implanted under the skin along their spine between their shoulders. At ~1 week post-surgery mice received an injection of 10^4^ *cfu* of *L*. *monocytogenes*, EGD strain. Each mouse’s movement/activity was recorded every 5 seconds and reported as accumulated events for every 20 minute period. Each tick mark on the X-axis represents an hour. Data shown represent the mean activity (#events) of four mice in each diet treatment group (n = 4/diet treatment group).
(TIF)

S2 Fig Impact of Dietary *S*. *frutescens* on Spontaneous Activity (i.e., sickness behavior) of Male Mice After an Experimental Infection with *E*. *coli*. Healthy male C57BL/6 weanling mice were fed experimental diets containing one of three doses of *S*. *frutescens* (i.e., 0, 0.25 or 1% by wt) for 3–4 wks. Mice were housed in pairs in a vivarium with a 12 hr light:dark cycle at a room temperature between 22-25°C and a relative humidity of 50–60% for the entirety of the study. One of each pair of mice had a mini-emitter surgically implanted under the skin along their spine between their shoulders. At ~1 week post-surgery mice received an injection of ~10^8^ *cfu* of *E*. *coli* K12 strain. Each mouse’s movement/activity was recorded every 5 seconds and reported as accumulated events for every 20 minute period. Each tick mark on the X-axis represents an hour. Data shown represent the mean activity (#events) of four mice in each diet treatment group (n = 4/diet treatment group).
(TIF)

S1 Table Impact of Dietary *S*. *frutescens* on Body, Liver, and Spleen Weight of Mice Following an *L*. *monocytogenes* challenge. Healthy female and male BALB/c weanling mice were fed experimental diets containing one of three doses of *S*. *frutescens* (i.e., 0, 0.25 or 1% by wt) for 3–4 wks. At ~7 wk of age, mice were weighed (i.e., pre-challenge), then injected intravenously with ~10^4^ *cfu* of *L*. *monocytogenes*, EGD strain. Three days following the challenge, mice were re-weighed (i.e., post-challenge), then humanely killed for the collection of liver and spleen, which were weighed and then homogenized for the subsequent enumeration of bacteria. All values are expressed as means ± SEM (n = 15–16 per dietary treatment group).
(DOCX)

S2 Table Impact of Dietary *S*. *frutescens* on Body, Liver, and Spleen Weight of Mice Following an *E*. *coli* challenge. Healthy female and male C57Bl/6 weanling mice were fed experimental diets containing one of three doses of *S*. *frutescens* (i.e., 0, 0.25 or 1% by wt) for 3–4 wks. At ~7 wk of age, mice were weighed (i.e., pre-challenge), then injected intravenously with ~10^8^ *cfu* of *E*. *coli*, K12 strain. Two days following the challenge, mice were re-weighed (i.e., post-challenge), then humanely killed for the collection of liver and spleen, which were weighed and then homogenized for the subsequent enumeration of bacteria. All values are expressed as means ± SEM (n = 15–16 per dietary treatment group).
(DOCX)

S3 Table Serum Cytokines and Chemokines from Mice 2 hours Following an Injection with Vehicle (PBS). For this study, male C57Bl/6 mice were fed experimental diets containing one of three doses of *S*. *frutescens* (0, 0.25 and 1% SF by wt) for 3 wks. Mice were injected at 1 min intervals with 1.0 mL of sterile PBS (i.e., vehicle controls for LPS challenged mice). Two hours following this injection blood was collected from mice and allowed to clot for 30 minutes at room temperature. Serum was collected by centrifugation and stored at -80°C until assayed for cytokines/chemokines using a commercial multiplex kit. Data shown are from eight mice from each diet treatment group (n = 8/trt); values represent means ± SEM (pg/mL). Diet intervention/treatment, as tested by ANOVA, failed to significantly affect any measured parameter (i.e., *p* > 0.05).
(DOCX)

S4 Table *Ex Vivo* LPS-induced Inflammatory Cytokine and Chemokine Production by Primary Macrophages Isolated from Mice Fed Differing Levels of *S*. *frutescens*. For this experiment, male C57BL/6 mice were fed experimental diets containing one of three doses of *S*. *frutescens* (0, 0.25 and 1% by wt) for 3–4 wks. Peritoneal macrophages were isolated 3 days following intraperitoneal injection with sterile thioglycolate broth. Adherent cells (i.e., >95% macrophages) were co-cultured with 100 ng/mL of LPS (from *E*. *coli* 0111:B4) and 24 h cell later culture supernatants were collected and subsequently diluted 10-fold in FBS-free DMEM, then analyzed for specific cytokines and chemokines using a commercial multiplex beads-based assay system. Data shown are from twelve mice from each diet treatment group (n = 11-12/trt); all values are LSmeans ± pooled SEM, expressed in pg/mL, unless otherwise indicated.
(DOCX)

S5 Table *Ex Vivo* Pam2CSK-induced Inflammatory Cytokine and Chemokine Production by Primary Macrophages Isolated from Mice Fed Differing Levels of *S*. *frutescens*. For this experiment, male C57BL/6 mice were fed experimental diets containing one of three doses of *S*. *frutescens* (0, 0.25 and 1% by wt) for 3–4 wks. Peritoneal macrophages were isolated 3 days following intraperitoneal injection with sterile thioglycolate broth. Adherent cells (i.e., >95% macrophages) were co-cultured with 5 ng of Pam2CSK and 24 h later cell culture supernatants were collected and subsequently diluted 10-fold in FBS-free DMEM, then analyzed for specific cytokines and chemokines using a commercial multiplex beads-based assay system. Data shown are from twelve mice from each diet treatment group (n = 12/trt); all values are LSmeans ± pooled SEM, expressed in pg/mL, unless otherwise indicated.
(DOCX)
