# Supplementary figures and images for: An Investigation into the Immunomodulatory Activities of Sutherlandia frutescens in Healthy Mice
Source: PLoS One. 2016 Aug 30;11(8):e0160994. doi: 10.1371/journal.pone.0160994 (PMC5004858; doi:10.1371/journal.pone.0160994)

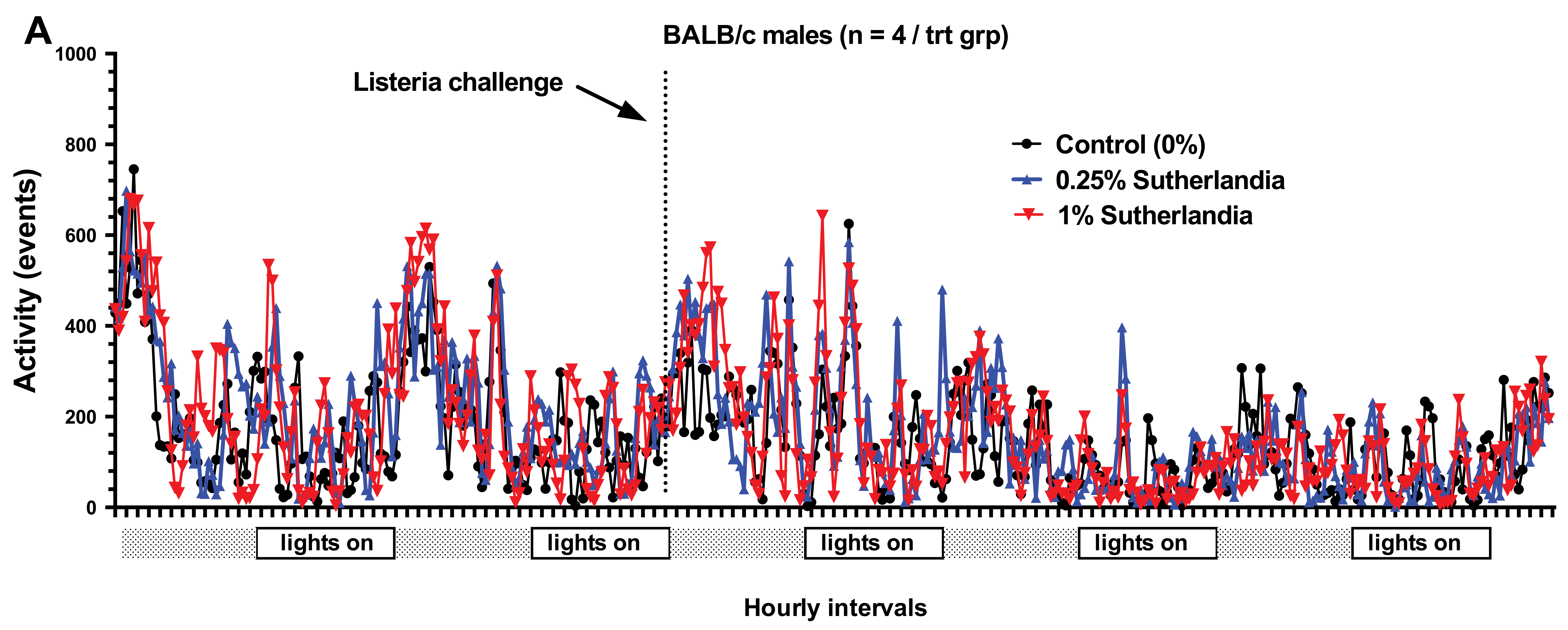

Supplement: S1 Fig — Healthy male BALB/c weanling mice were fed experimental diets containing one of three doses of S. frutescens (i.e., 0, 0.25 or 1% by wt) for 3–4 wks. Mice were housed in pairs in a vivarium with a 12 hr light:dark cycle at a room temperature between 22-25°C and a relative humidity of 50–60% for the entirety of the study. One of each pair of mice had a mini-emitter surgically implanted under the skin along their spine between their shoulders. At ~1 week post-surgery mice received an injection of 104 cfu of L. monocytogenes, EGD strain. Each mouse’s movement/activity was recorded every 5 seconds and reported as accumulated events for every 20 minute period. Each tick mark on the X-axis represents an hour. Data shown represent the mean activity (#events) of four mice in each diet treatment group (n = 4/diet treatment group). (TIF) [file pone.0160994.s001.tif]

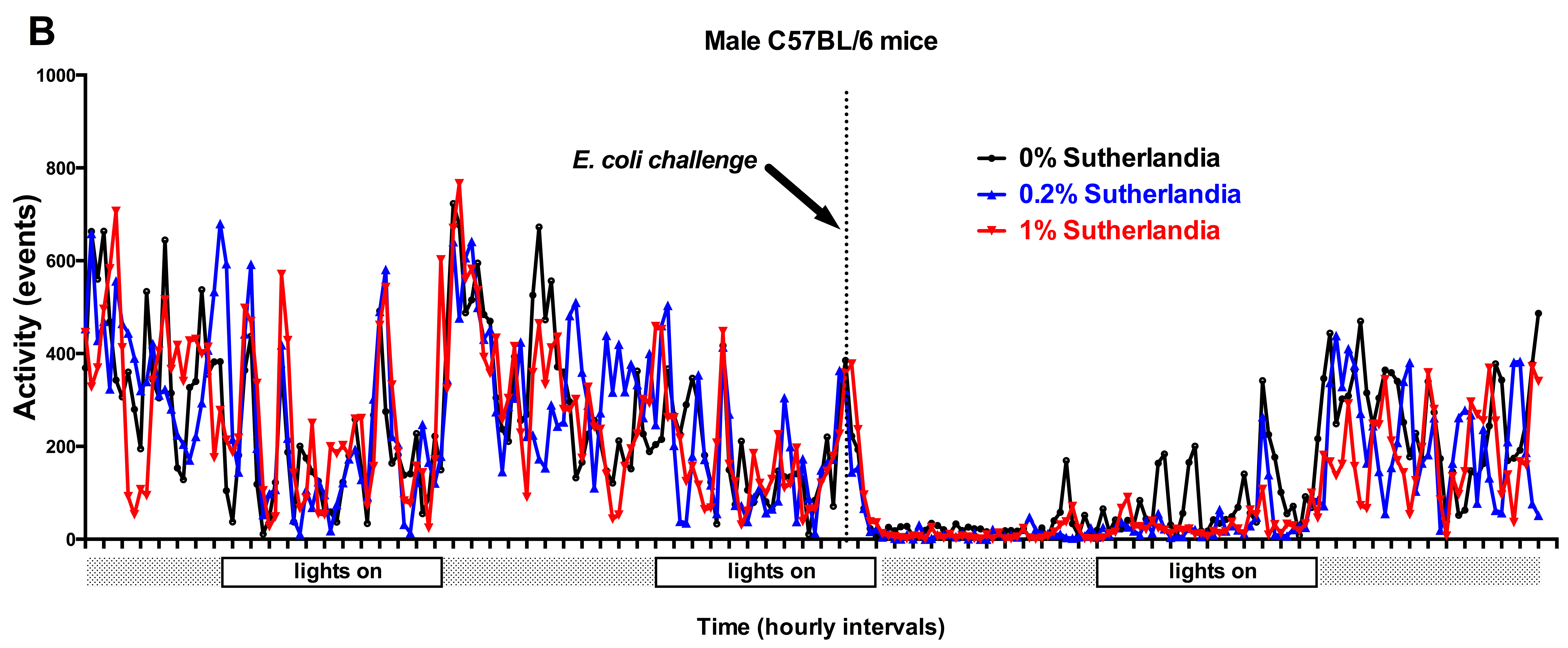

Supplement: S2 Fig — Healthy male C57BL/6 weanling mice were fed experimental diets containing one of three doses of S. frutescens (i.e., 0, 0.25 or 1% by wt) for 3–4 wks. Mice were housed in pairs in a vivarium with a 12 hr light:dark cycle at a room temperature between 22-25°C and a relative humidity of 50–60% for the entirety of the study. One of each pair of mice had a mini-emitter surgically implanted under the skin along their spine between their shoulders. At ~1 week post-surgery mice received an injection of ~108 cfu of E. coli K12 strain. Each mouse’s movement/activity was recorded every 5 seconds and reported as accumulated events for every 20 minute period. Each tick mark on the X-axis represents an hour. Data shown represent the mean activity (#events) of four mice in each diet treatment group (n = 4/diet treatment group). (TIF) [file pone.0160994.s002.tif]
