# Supplementary material for: An Investigation into the Immunomodulatory Activities of Sutherlandia frutescens in Healthy Mice
Source: PLoS One. 2016 Aug 30;11(8):e0160994. doi: 10.1371/journal.pone.0160994 (PMC5004858; doi:10.1371/journal.pone.0160994)
Supplement: S1 Table — Healthy female and male BALB/c weanling mice were fed experimental diets containing one of three doses of S. frutescens (i.e., 0, 0.25 or 1% by wt) for 3–4 wks. At ~7 wk of age, mice were weighed (i.e., pre-challenge), then injected intravenously with ~104 cfu of L. monocytogenes, EGD strain. Three days following the challenge, mice were re-weighed (i.e., post-challenge), then humanely killed for the collection of liver and spleen, which were weighed and then homogenized for the subsequent enumeration of bacteria. All values are expressed as means ± SEM (n = 15–16 per dietary treatment group). (DOCX) [file pone.0160994.s003.docx]

**S1 Table. Impact of Dietary *S. frutescens* on Body, Liver, and Spleen Weight of Mice Following an *L. monocytogenes* challenge.*^a^***

|  | **Experimental Diet Treatments** | | | *p*-value |
| --- | --- | --- | --- | --- |
|  | **Control** | **0.25% SF** | **1.0% SF** |  |
| **Female BALB/c** | | | | |
| Pre-challenge body weight (g) | 18.6 ± 0.4 | 18.4 ± 0.3 | 18.5 ± 0.6 | ns *^b^* |
| Post-challenge body weight (g) | 17.1 ± 0.5 | 16.4 ± 0.4 | 16.8 ± 0.3 | ns |
| Liver (mg) | 867 ± 26 | 894 ± 25 | 886 ± 23 | ns |
| Spleen (mg) | 147 ± 10 | 139 ± 8 | 138 ± 6 | ns |
| **Male BALB/c** | | | | |
| Pre-challenge body weight (g) | 24.9 ± 0.6 | 24.9 ± 0.3 | 24.2 ± 0.4 | ns |
| Post-challenge body weight (g) | 23.5 ± 0.5 | 23.5 ± 0.5 | 22.6 ± 0.7 | ns |
| Liver (mg) | 1154 ± 34 | 1221 ± 46 | 1150 ± 40 | ns |
| Spleen (mg) | 168 ± 7 | 172 ± 7 | 160 ± 7 | ns |

***^a^*** Healthy female and male BALB/c weanling mice were fed experimental diets containing one of three doses of *S. frutescens* (i.e., 0, 0.25 or 1% by wt) for 3-4 wks. At ~7 wk of age, mice were weighed (i.e., pre-challenge), then injected intravenously with ~10^4^ *cfu* of *L. monocytogenes,* EGD strain. Three days following the challenge, mice were re-weighed (i.e., post-challenge), then humanely killed for the collection of liver and spleen, which were weighed and then homogenized for the subsequent enumeration of bacteria. All values are expressed as means ± SEM (n =15-16 per dietary treatment group). *^b^*ns = not significant (*p-*value > 0.05).
