# Supplementary material for: An Investigation into the Immunomodulatory Activities of Sutherlandia frutescens in Healthy Mice
Source: PLoS One. 2016 Aug 30;11(8):e0160994. doi: 10.1371/journal.pone.0160994 (PMC5004858; doi:10.1371/journal.pone.0160994)
Supplement: S2 Table — Healthy female and male C57Bl/6 weanling mice were fed experimental diets containing one of three doses of S. frutescens (i.e., 0, 0.25 or 1% by wt) for 3–4 wks. At ~7 wk of age, mice were weighed (i.e., pre-challenge), then injected intravenously with ~108 cfu of E. coli, K12 strain. Two days following the challenge, mice were re-weighed (i.e., post-challenge), then humanely killed for the collection of liver and spleen, which were weighed and then homogenized for the subsequent enumeration of bacteria. All values are expressed as means ± SEM (n = 15–16 per dietary treatment group). (DOCX) [file pone.0160994.s004.docx]

**S2 Table. Impact of Dietary *S. frutescens* on Body, Liver, and Spleen Weight of Mice Following an *E. coli* challenge.*^a^***

|  | **Experimental Diet Treatments** | | | *p*-value |
| --- | --- | --- | --- | --- |
|  | **Control** | **0.25% SF** | **1.0% SF** |  |
| **Female C57Bl/6 mice** | | | | |
| Pre-challenge body weight (g) | 19.5 ± 0.5 | 19.6 ± 0.3 | 19.8 ± 0.4 | ns *^b^* |
| Post-challenge body weight (g) | 17.7 ± 0.6 | 17.5 ± 0.7 | 17.5 ± 0.5 | ns |
| Liver (mg) | 778 ± 37 | 877 ± 23 | 868 ± 24 | ns |
| Spleen (mg) | 109 ± 7 | 103 ± 5 | 113 ± 6 | ns |
| **Male C57Bl/6 mice** | | | | |
| Pre-challenge body weight (g) | 26.2 ± 0.4 | 26.6 ± 0.5 | 25.6 ± 0.4 | ns |
| Post-challenge body weight (g) | 23.7 ± 0.6 | 24.0 ± 0.3 | 23.1 ± 0.5 | ns |
| Liver (mg) | 1085 ± 53 | 1062 ± 45 | 1030 ± 33 | ns |
| Spleen (mg) | 121 ± 6 | 115 ± 5 | 111 ± 4 | ns |

***^a^*** Healthy female and male C57Bl/6 weanling mice were fed experimental diets containing one of three doses of *S. frutescens* (i.e., 0, 0.25 or 1% by wt) for 3-4 wks. At ~7 wk of age, mice were weighed (i.e., pre-challenge), then injected intraperitoneally with ~10^8^ *cfu* of *E. coli* K12 strain. Two days following the challenge, mice were re-weighed (i.e., post-challenge), then humanely killed for the collection of liver and spleen, which were weighed and then homogenized for the subsequent enumeration of bacteria. All values are expressed as means ± SEM (n =15-16 per dietary treatment group). *^b^*ns = not significant (*p-*value > 0.05).
