# Supplementary material for: An Investigation into the Immunomodulatory Activities of Sutherlandia frutescens in Healthy Mice
Source: PLoS One. 2016 Aug 30;11(8):e0160994. doi: 10.1371/journal.pone.0160994 (PMC5004858; doi:10.1371/journal.pone.0160994)
Supplement: S3 Table — For this study, male C57Bl/6 mice were fed experimental diets containing one of three doses of S. frutescens (0, 0.25 and 1% SF by wt) for 3 wks. Mice were injected at 1 min intervals with 1.0 mL of sterile PBS (i.e., vehicle controls for LPS challenged mice). Two hours following this injection blood was collected from mice and allowed to clot for 30 minutes at room temperature. Serum was collected by centrifugation and stored at -80°C until assayed for cytokines/chemokines using a commercial multiplex kit. Data shown are from eight mice from each diet treatment group (n = 8/trt); values represent means ± SEM (pg/mL). Diet intervention/treatment, as tested by ANOVA, failed to significantly affect any measured parameter (i.e., p > 0.05). (DOCX) [file pone.0160994.s005.docx]

**S3 Table. Serum Cytokines and Chemokines from Mice 2 hours Following an Injection with Vehicle (PBS).*^a^***

|  | **Experimental Diet Treatments** | | |  |
| --- | --- | --- | --- | --- |
| **Analyte*^b^*** | **Control** | **0.25% SF** | **1% SF** | **MDC***^c^* |
| TNF-α | 5.3 ± 1.6 | 5.7 ± 2.4 | 4.9 ± 1.6 | 1.6 |
| IL-1α | 244 ± 45 | 246 ± 57 | 268 ± 55 | 4.2 |
| IL-1β | 10 ± 2 | 9 ± 2 | 10 ± 3 | 3.2 |
| IL-6 | 45 ± 17 | 36 ± 8 | 116 ± 67 | 0.6 |
| IL-10 | 5 ± 1 | 5 ± 1 | 10 ± 5 | 3.4 |
| IL-12p40 | 9 ± 2 | 11 ± 3 | 9 ± 1 | 3.2 |
| IL-12p70 | 6 ± 1 | 7 ± 3 | 9 ± 5 | 3.6 |
| IFN-γ | 3 ± 1 | 5 ± 2 | 3 ± 1 | 0.8 |
| G-CSF | 895 ± 390 | 560 ± 78 | 777 ± 316 | 3.2 |
| GM-CSF | 24 ± 4 | 28 ± 3 | 18 ± 4 | 2.8 |
| MCP-1 (CCL2) | 68 ± 22 | 38 ± 8 | 48 ± 16 | 3.6 |
| MIP-1α (CCL3) | 48 ± 9 | 36 ± 8 | 57 ± 20 | 5.0 |
| MIP-1β (CCL4) | 41 ± 14 | 37 ± 5 | 25 ± 8 | 8.4 |
| RANTES (CCL5) | 49 ± 6 | 47 ± 5 | 45 ± 5 | 2.0 |
| KC (CXCL1) | 247 ± 97 | 88 ± 12 | 225 ± 81 | 1.8 |
| MIP-2α (CXCL2) | 127 ± 10 | 127 ± 10 | 131 ± 18 | 1.2 |
| IP-10 (CXCL10) | 227 ± 32 | 213 ± 17 | 194 ± 9 | 2.4 |

*^a^* For this study, male C57Bl/6 mice were fed experimental diets containing one of three doses of *S. frutescens* (0, 0.25 and 1% SF, by wt) for 3 wks. Mice were injected at 1 min intervals with 1.0 mL of sterile PBS (i.e., vehicle controls for LPS challenged mice). Two hours following this injection blood was collected from mice and allowed to clot for 30 minutes at room temperature. Serum was collected by centrifugation and stored at -80° C until assayed for cytokines/chemokines using a commercial multiplex kit. Data shown are from eight mice from each diet treatment group (n = 8/trt); values represent means ± SEM (pg/mL). Diet intervention/treatment, as tested by ANOVA, failed to significantly affect any measured analyte (i.e., *p* > 0.05).

*^b^* Abbreviations (refer to Tables 2 and 3).

*^c^* MDC = “minimal detection concentration” according to the kit’s manufacturer and adjusted to account for the 1:2 dilution recommended for sera.
