# Supplementary material for: An Investigation into the Immunomodulatory Activities of Sutherlandia frutescens in Healthy Mice
Source: PLoS One. 2016 Aug 30;11(8):e0160994. doi: 10.1371/journal.pone.0160994 (PMC5004858; doi:10.1371/journal.pone.0160994)
Supplement: S4 Table — For this experiment, male C57BL/6 mice were fed experimental diets containing one of three doses of S. frutescens (0, 0.25 and 1% by wt) for 3–4 wks. Peritoneal macrophages were isolated 3 days following intraperitoneal injection with sterile thioglycolate broth. Adherent cells (i.e., >95% macrophages) were co-cultured with 100 ng/mL of LPS (from E. coli 0111:B4) and 24 h cell later culture supernatants were collected and subsequently diluted 10-fold in FBS-free DMEM, then analyzed for specific cytokines and chemokines using a commercial multiplex beads-based assay system. Data shown are from twelve mice from each diet treatment group (n = 11-12/trt); all values are LSmeans ± pooled SEM, expressed in pg/mL, unless otherwise indicated. (DOCX) [file pone.0160994.s006.docx]

**S4 Table. *Ex Vivo* LPS-induced Inflammatory Cytokine and Chemokine Production by Primary Macrophages Isolated from Mice Fed Differing Levels of *S. frutescens*.*^a^***

|  | **Experimental Diet Treatments** | | |  |
| --- | --- | --- | --- | --- |
| **Analyte*^b^*** | **Control** | **0.25% SF** | **1% SF** | ***p*-value*^c^*** |
| TNF-α ***^d^*** | 17.8 ± 6.3 | 28.0 ± 6.3 | 19.2 ± 6.0 | 0.53 |
| IL-1α | 2694 ± 324 | 1789 ± 313*^e^* | 2087 ± 313 | 0.08 |
| IL-1β | 454 ± 81 | 554 ± 81 | 374 ± 81 | 0.86 |
| IL-6 ***^d^*** | 11.9 ± 1.2 | 10.7 ± 1.2 | 10.6 ± 1.2 | 0.85 |
| IL-10 | 351 ± 90 | 320 ± 86 | 362 ± 90 | 0.42 |
| IL-12p40 | 1078 ± 218 | 1518 ± 218 | 1408 ± 209 | 0.55 |
| IL-12p70 | 288 ± 36 | 269 ± 35 | 228 ± 36 | 0.77 |
| IL-13 | 147 ± 32 | 151 ± 32 | 123 ± 32 | 0.68 |
| IL-15 | 283 ± 106 | 288 ± 102 | 409 ± 102 | 0.91 |
| IL-17 | 43 ± 26 | 114 ± 26 | 41 ± 26 | 0.74 |
| IFN-γ | 31 ± 10 | 25 ± 10 | 22 ± 10 | 0.69 |
| G-CSF ***^d^*** | 3179 ± 482 | 3598 ± 466 | 3643 ± 460 | 0.84 |
| GM-CSF | 287 ± 98 | 415 ± 98 | 334 ± 98 | 0.31 |
| MCP-1 (CCL2) ***^d^*** | 42.4 ± 5.3 | 40.7 ± 5.1 | 45.1 ± 5.1 | 0.93 |
| MIP-1α (CCL3) ***^d^*** | 5.8 ± 0.8 | 5.7 ± 0.7 | 5.9 ± 0.7 | 0.45 |
| MIP-1β (CCL4) ***^d^*** | 11.0 ± 0.7 | 10.4 ± 0.7 | 12.2 ± 0.7 | 0.76 |
| RANTES (CCL5) ***^d^*** | 33.3 ± 8.3 | 36.9 ± 8.0 | 33.9 ± 8.3 | 0.78 |
| KC (CXCL1) ***^d^*** | 194 ± 11 | 188 ± 11 | 198 ± 11 | 0.53 |
| MIP-2α (CXCL2) ***^d^*** | 31.4 ± 3.0 | 32.8 ± 3.0 | 33.9 ± 3.0 | 0.46 |
| IP-10 (CXCL10) | 11.4 ± 1.3 | 10.9 ± 1.3 | 11.9 ± 1.3 | 0.44 |

^a^ For this experiment, male C57BL/6 mice were fed experimental diets containing one of three doses of *S. frutescens* (0, 0.25 and 1% by wt) for 3-4 wks. Peritoneal macrophages were isolated 3 days following intraperitoneal injection with sterile thioglycolate broth. Adherent cells (i.e., >95% macrophages) were co-cultured with 100 ng/mL of LPS (from *E. coli* 0111:B4) and 24 h later cell culture supernatants were collected and subsequently diluted 10-fold in FBS-free DMEM, then analyzed for specific cytokines and chemokines using a commercial multiplex beads-based assay system. Data shown are from twelve mice from each diet treatment group (n = 12/trt); all values are LSmeans ± pooled SEM, expressed in pg/mL, unless otherwise indicated.

*^b^* The impact of the diet intervention/treatment was tested in SAS by ANOVA, using contrast with interaction between the two independent trials/experiments with the main effect *p-*value shown in the 4^th^ column.

^c^ Abbreviations: refer to Tables 2 and 3.

*^d^* Means reported in ng/mL.

*^e^* Different from control (*p* < 0.05).
