# Supplementary material for: An Investigation into the Immunomodulatory Activities of Sutherlandia frutescens in Healthy Mice
Source: PLoS One. 2016 Aug 30;11(8):e0160994. doi: 10.1371/journal.pone.0160994 (PMC5004858; doi:10.1371/journal.pone.0160994)
Supplement: S5 Table — For this experiment, male C57BL/6 mice were fed experimental diets containing one of three doses of S. frutescens (0, 0.25 and 1% by wt) for 3–4 wks. Peritoneal macrophages were isolated 3 days following intraperitoneal injection with sterile thioglycolate broth. Adherent cells (i.e., >95% macrophages) were co-cultured with 5 ng of Pam2CSK and 24 h later cell culture supernatants were collected and subsequently diluted 10-fold in FBS-free DMEM, then analyzed for specific cytokines and chemokines using a commercial multiplex beads-based assay system. Data shown are from twelve mice from each diet treatment group (n = 12/trt); all values are LSmeans ± pooled SEM, expressed in pg/mL, unless otherwise indicated. (DOCX) [file pone.0160994.s007.docx]

**S5 Table. *Ex Vivo* Pam2CSK-induced Inflammatory Cytokine and Chemokine Production by Primary Macrophages Isolated from Mice Fed Differing Levels of *S. frutescens*.*^a^***

|  | **Experimental Diet Treatments** | | |  |
| --- | --- | --- | --- | --- |
| **Analyte*^b^*** | **Control** | **0.25% SF** | **1% SF** | ***p*-value*^c^*** |
| TNF-α ***^d^*** | 24.7 ± 6.3 | 30.9 ± 6.1 | 24.9 ± 6.1 | 0.78 |
| IL-1α | 2614 ± 351 | 2478 ± 339 | 2506 ± 339 | 0.78 |
| IL-1β | 544 ± 128 | 588 ± 119 | 286 ± 119 | 0.22 |
| IL-6 ***^d^*** | 25.9 ± 3.0 | 24.1 ± 2.9 | 19.1 ± 2.9 | 0.46 |
| IL-10 | 390 ± 102 | 327 ± 71 | 387 ± 71 | 0.91 |
| IL-12p40 | 877 ± 165 | 887 ± 153 | 881 ± 153 | 0.97 |
| IL-12p70 | 382 ± 52 | 304 ± 50 | 241 ± 50 | 0.09 |
| IL-13 | 306 ± 102 | 247 ± 99 | 416 ± 102 | 0.84 |
| IL-15 | 117 ± 31 | 49 ± 30 | 49 ± 30 | 0.09 |
| IL-17 | 24.0 ± 7.2 | 12.5 ± 6.9 | 11.5 ± 7.2 | 0.18 |
| IFN-γ | 113 ± 25 | 122 ± 24 | 62 ± 24 | 0.40 |
| G-CSF ***^d^*** | 9.1 ± 2.3 | 11.7 ± 2.2 | 8.1 ± 2.2 | 0.94 |
| GM-CSF | 461 ± 110 | 580 ± 106 | 427 ± 102 | 0.99 |
| MCP-1 (CCL2) ***^d^*** | 35.2 ± 3.6 | 35.8 ± 3.5 | 33.8 ± 3.5 | 0.53 |
| MIP-1α (CCL3) ***^d^*** | 7.8 ± 1.0 | 6.3 ± 0.9 | 5.6 ± 0.9 | 0.55 |
| MIP-1β (CCL4) ***^d^*** | 10.5 ± 1.4 | 9.7 ± 1.4 | 8.6 ± 1.4 | 0.59 |
| RANTES (CCL5) ***^d^*** | 61 ± 29 | 14 ± 27 | 8 ± 28 | 0.29 |
| KC (CXCL1) ***^d^*** | 222 ± 10 | 232 ± 9 | 224 ± 10 | 0.78 |
| MIP-2α (CXCL2) ***^d^*** | 159 ± 8 | 159 ± 8 | 158 ± 8 | 0.66 |
| IP-10 (CXCL10) | 622 ± 164 | 394 ± 152 | 330 ± 157 | 0.20 |

^a^ For this experiment, male C57BL/6 mice were fed experimental diets containing one of three doses of *S. frutescens* (0, 0.25 and 1% by wt) for 3-4 wks. Peritoneal macrophages were isolated 3 days following intraperitoneal injection with sterile thioglycolate broth. Adherent cells (i.e., >95% macrophages) were co-cultured with 5 ng of Pam2CSK and 24 h later cell culture supernatants were collected and subsequently diluted 10-fold in FBS-free DMEM, then analyzed for specific cytokines and chemokines using a commercial multiplex beads-based assay system. Data shown are from twelve mice from each diet treatment group (n = 12/trt); all values are LSmeans ± pooled SEM, expressed in pg/mL, unless otherwise indicated.

*^b^* The impact of the diet intervention/treatment was tested in SAS by ANOVA, using contrast with interaction between the two independent trials/experiments with the main effect *p-*value shown in the 4^th^ column.

^c^ Abbreviations: refer to Tables 2 and 3.

*^d^* Means reported in ng/mL.
